# The epidemiology of and management of pediatric patients with head trauma: a hospital-based study from Southern Sweden

**DOI:** 10.1186/s13049-022-01055-9

**Published:** 2022-12-09

**Authors:** Ali Al Mukhtar, Henrik Bergenfeldt, Marcus Edelhamre, Tomas Vedin, Per-Anders Larsson, Stefan Öberg

**Affiliations:** 1 Departments of Surgery, Skåne’s University Hospital, Carl-Bertil Laurells Gata 9, 214 28 Malmö, Sweden; 2 Helsingborg Hospital, Helsingborg, Sweden; 3 Skaraborg Hospital, Skövde, Sweden; 4 Department of Clinical Sciences, Lund University, Lund, Sweden

**Keywords:** Pediatric traumatic brain injury, Isolated head trauma, Multitrauma, Computed tomography

## Abstract

**Background:**

Traumatic brain injury (TBI) is a common cause of morbidity and mortality in children worldwide. In Scandinavia, the epidemiology of pediatric head trauma is poorly documented. This study aimed to investigate and compare the epidemiology and management of pediatric patients with isolated head trauma (IHT) and head trauma in connection with multitrauma (MHT).

**Methods:**

We conducted a retrospective review of medical records of patients < 18 years of age who attended any of the five emergency departments (ED) in Scania County in Sweden in 2016 due to head trauma. Clinical data of patients with IHT were analyzed and compared with those of patients with MHT.

**Results:**

We identified 5046 pediatric patients with head trauma, 4874 with IHT and 186 with MHT, yielding an incidence of ED visits due to head trauma of 1815/100,000 children/year. There was male predominance, and the median age was four years. Falls were the dominating trauma mechanism in IHT patients, while motor vehicle accidents dominated in MHT patients. The frequencies of CT head-scans, ward admissions and intracranial injuries (ICI) were 5.4%, 11.1% and 0.7%, respectively. Four patients (0.08%) required neurosurgical intervention. The relative risks for CT-scans and admissions to a hospital ward and ICI were 10, 4.5 and 19 times higher for MHT compared with IHT patients.

**Conclusion:**

Head trauma is a common cause of ED visits in our study. Head-CTs and ICIs were less frequent than in previous studies. MHT patients had higher rates of CT-scans, admissions, and ICIs than IHT patients, suggesting that they are separate entities that should ideally be managed using different guidelines to optimize the use of CT-scans of the head.

## Introduction

Traumatic brain injury (TBI) is defined as an injury caused by an external force to the head which causes an alteration in brain function or other evidence of brain pathology [1]. TBI is an important global health issue as it is one of the most common causes of morbidity and mortality in children worldwide [2–4]. Epidemiological studies describing the incidence and outcomes of TBI are necessary for increasing public awareness to enable targeted prevention and to improve the diagnosis and treatment of patients with TBI.

Although there are several reports on pediatric TBI globally and in Europe in particular, the epidemiology of pediatric TBI remains unclear as differences in the definition of TBI and patient selections make available data difficult to interpret [1, 5]. In England and Wales, 1.4 million people attend emergency departments (ED) yearly due to recent head trauma. Between 33 and 50% of these are children younger than 15 years [6]. In the United States, a population-based study reported approximately 2356 TBI-related ED visits, 68 TBI-related hospitalizations, and 6 TBI-related deaths per 100,000 children 15 years or younger [7].

Head trauma in children is a frequent cause of ED visits and hospitalizations globally [5], but the fact that only a small fraction of children with head trauma have intracranial injuries (ICIs) [4] leads to diagnostic challenges for physicians who handle children with head trauma. Computed tomography scan (CT-scan) of the head is the gold standard for identifying patients with TBI [4, 8] but CT-scanning should ideally only be performed in carefully selected patient groups as ionizing radiation is associated with an increased risk for leukemia and brain tumors[9]. Therefore, the primary objective in the emergency management of children with head trauma is to reliably diagnose the small fraction of children with ICI, but also to avoid excessive radiation caused by CT-scanning.

Tailored Clinical Decision Rules (CDR) can be utilized to manage children with head trauma with the aim of finding all children with ICI and reducing the number of negative CT-scans. Such CDRs include the Pediatric Emergency Care Applied Research Network (PECARN) rule and the Scandinavian Neurotrauma Committee (SNC)’s guidelines, among others [4, 10–12]. Validation studies of these CDRs have shown CT-scanning rates ranging between 8 and 30% [8, 13, 14]. Considering the low frequency of ICI in pediatric patients with head trauma, the relatively high CT-scanning rates possibly imply an excessive use of CT-scanning that hypothetically may be explained by the inclusion of children with isolated head trauma (IHT) as well as children with head trauma in connection with multitrauma (MHT) in these CDRs.

Head trauma can be either isolated or part of a multitrauma. Due to the high energy level in many multitraumas, these patients often sustain several and more severe injuries, and the risk for ICI is likely higher than in patients with IHT. Advanced Trauma Life Support (ATLS®) guidelines are used widely and globally to manage multitrauma patients with or without head injury [15]. In the most recent edition of the ATLS guidelines [15], employment of the PECARN guidelines are recommended in the management of children at low risk for brain injury in a multitrauma setting. Consequently, CDRs, such as the PECARN rule, are used in the management of both IHT and MHT patients.

This hospital-based study aimed to assess the epidemiology and the management of children with head trauma by assessing and comparing the rates of ED visits, the rates of CT-scans, hospital admissions and ICIs in children with IHT and MHT, attending any of the EDs in Scania County.

## Methods

This study was performed as a retrospective review of medical records of pediatric patients with head trauma attending any of the five EDs in Scania County in Sweden from 1 January 2016 to 31 December 2016. Patients with insufficient patient records as well as patients with isolated facial trauma without trauma to the neurocranium were not included in the study. In 2016, Scania county had a catchment area of 278,773 children < 18 years of age [16, 17]. We obtained the medical records of all children who were registered in the local electronic registration system with head trauma or multitrauma. This inclusion method was preferred over patient selection with international classification of disease (ICD) codes, as it enabled us to identify all patients with suspected head trauma and/or multitrauma and manually exclude those who did not meet the inclusion criteria. The Berlin definition was used to identify patients with multitrauma [18].

We chose not to perform injury severity scores for comparisons between groups of patients with IHT and MHT, as the level of trauma energy typically is higher in patients with multitrauma. We believe the use of injury severity scores would provide no new or useful information regarding patient management, especially in patients with IHT. Instead, we chose to compare groups of patients based on history and clinical findings, as we believe this information provides better insight in factors that are clinically important in the management of children with head trauma.

The clinical information collected for patients included patient characteristics, the performance of a CT-scan of the head, admission to a hospital ward or intensive care unit (ICU), and if they were found to have an ICI and the need for neurosurgical intervention (NI). ICI was defined as evidence of intracranial bleeding and/or skull fracture on CT-scan/radiology, and NI included intracranial pressure monitoring, the elevation of skull fractures and craniotomies. We used the patients’ medical records to identify subjects who died during the study period and whether the cause of death was due to TBI. In Sweden, every death is reported within 24 h to the Swedish Tax Agency, and all medical registries are updated on a routine basis at least weekly to match the Agency registries.

To reduce the risk of information bias during data collection, we followed the guidelines for retrospective medical record reviews by Vassar and Holzman [19]. A proforma protocol detailing the collection process of the parameters was created, tested, and revised. There was a total of five data collectors. All five were trained to collect data using the proforma protocol prior to actual data collection. Individual interpretation uncertainties were discussed within the research team before recording.

### Statistics

Shapiro–Wilk’s formula and histograms were used to investigate data distribution. As the data were not normally distributed, results were presented using median and 25–75th percentiles. The Mann–Whitney U test was used to compare continuous data between two groups. Comparisons of proportions were made using the Chi-square test, and relative risks and 95% confidence intervals (CI) were calculated. A *p*-value of less than 0.05 was considered to represent statistical significance. All statistical analyses were performed using IBM SPSS Statistics 26®.

## Results

During the study period, we identified 5046 pediatric patients who attended any of the five EDs in Scania County due to IHT. Additionally, we identified 387 children with multitrauma, 186 of whom had suspected trauma to the head. We excluded 172 of the patients excluded due to insufficient patient records. Consequently, the study population consisted of 5060 pediatric patients with head trauma, 186 patients with MHT and 4874 patients with IHT. This resulted in an overall incidence of ED visit for head trauma of 1815 per 100,000 children and year and the incidence of IHT was 1748 per 100,000 children and year.

**Fig. 1:**
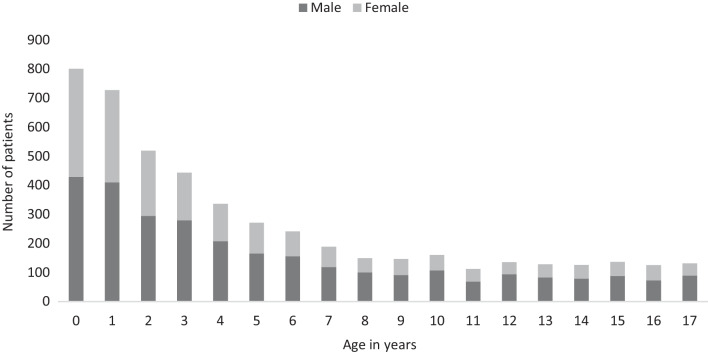
Age and gender distribution of children attending the emergency department due to isolated head trauma in Scania County (n = 4874)

**Fig. 2:**
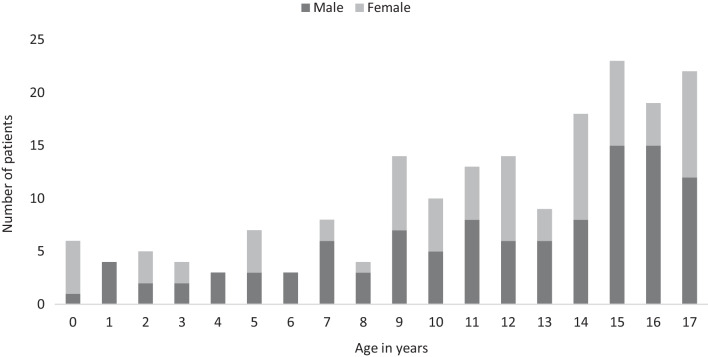
Age and gender distribution of multitrauma children with head trauma attending the emergency department in Scania County (n = 186)

The median age of patients with head trauma was 4 (1–10) years. Patients with IHT were significantly younger than MHT patients (3.0 (1.0–8.0) vs. 12.0 (9.0–15.0) years, *p* < 0.001). There was a male predominance in patients with IHT (60.2%) as well as in patients with MHT (58.6%) (Figs. 1 and 2).

Trauma mechanisms for patients with IHT and MHT are shown in (Table 1). The most common trauma mechanisms in patients with IHT were falls (64%) and collisions with an object or person (17.8%), while motor vehicle accidents (MVA) were relatively rare (1.2%). This contrasted with the trauma mechanisms in patients with MHT, in whom MVAs (39.2%) and falls (33.9%) were the dominating trauma mechanisms.

**Table 1:** Trauma mechanisms in pediatric patients attending emergency departments in Scania County due to isolated trauma to the head and head trauma in connection with multitrauma

	Patients with isolated headtrauma (n = 4874)	Multirauma patiens with head trauma (n = 186)
All falls	3123 (64.0%)	63 (33.9%)
Fall from ground level 0–50 cm	1334 (27.4%)	8 (4.3%)
Fall 51–90 cm	774 (15.9%)	12 (6.5%)
Fall 91–149 cm	294 (6.0%)	14 (7.5%)
Fall 150 cm–3 m	221 (4.5%)	22 (11.8%)
Fall > 3 m	7 (0.1%)	3 (1.6%)
Fall from unknown height	493 (10.1%)	4 (2.2%)
Collision with object or person	867 (17.8%)	11 (5.9%)
Hit with flying or falling object	196 (4.0%)	1 (0.5%)
Abuse without weapon	136 (2.8%)	1 (0.5%)
Abuse with weapon	21 (0.4%)	2 (1.1%)
Motor vehicle accident	58 (1.2%)	73 (39.2%)
Bicycle accident	181 (3.7%)	14 (7.5%)
Fall from skateboard or similar objects	118 (2.4%)	0 (0.0%)
Horseback riding accident	80 (1.6%)	21 (11.3%)
Unknown trauma mechanism	94 (1.9%)	0 (0.0%)

CT-scans of the head were performed in 5.4% of the study population (Table 2). The rate of CT-scans of the head and the rates of admissions to a hospital ward or an ICU were significantly higher in children with MHT compared with children with IHT. Similarly, children with MHT had a 10 times higher relative risk for undergoing CT-scans of the head, and 4.5- and 14-times higher risks for being admitted to a hospital ward or an ICU.

**Table 2:** Clinical findings and management in pediatric patients attending any of emergency departments in Scania County due to isolated trauma to the head and head trauma in connection with multitrauma

	All patients with head trauma (n = 5060)	Patients with isolated head trauma (n = 4874)	Multitrauma patients with head trauma (n = 186)	Relative risk* (95% CI)
CT-scans of the head	272(5.4%)	196 (4.0%)	76 (40.9%)**	10.2 (8.1–12.7)
Hospital admissions	564 (11.1%)	481 (9.9%)	83 (44.6%)**	4.5 (3.8–5.4)
CT-scan of the head and admission	125 (2.5%)	82 (1.7%)	43 (23.1%)**	13.7 (9.8–19.3)
Admission to ICU	17 (0.3%)	3 (0.06%)	14 (7.5%)**	122.3 (35.4–421.9)
ICI	33 (0.7%)	19 (0.4%)	14 (7.5%)**	19.3 (9.8–37.9)
Neurosurgical intervention	4 (0.08%)	1 (0.02%)	3 (1.6%)**	78.6 (8.2–752.1)

CT = Computed tomography, ICU = Intensive care unit, ICI = Intracranial injury

*Relative risk for patients with head trauma in connection with multitrauma compared with patients with isolated trauma to the head

**p-value < 0.001 compared with patients with isolated head trauma

ICI was found in 33 (0.7%) patients of the study population, which resulted in an overall incidence of 11.8 per 100,000 children and year (Table 2). In patients with IHT, ICI was a relatively rare event that was found in 0.4% of the patients, which contrasted with patients with MHT, in whom 7.5% of the patients were found to have an ICI. The relative risks for ICI and NI were 19.3 and 78.6 times higher in the MHT patients. In IHT patients with ICI, 18 patients had skull fractures, and nine patients were found to have intracranial bleedings. In the 14 MHT patients with ICI, skull fractures were found in nine patients and intracranial bleedings in 10 patients. Four patients required NI, three of whom were patients with MHT. One of the MHT patients required an elevation of depressed skull fracture while subdural hematomas were evacuated in two of them, and all three MHT patients required intracranial pressure monitoring. The IHT patient who required NI underwent an elevation of a depressed skull fracture. No patient was discharged from an ED to subsequently return to the ED to be diagnosed with an ICI, and no patients died during the study period.

## Discussion

This study is the largest epidemiological study of pediatric head trauma in Scandinavia, including more than 5000 children. It showed that pediatric head trauma is a common cause of ED visits in southern Sweden. The rates of CT-scanning and admissions and the incidence of ICI were lower than those reported in studies conducted outside of Scandinavia [4, 8, 20]. MHT patients had a significantly higher rate of ICI, more frequently underwent CT-scans, and had higher hospital admission rates than pediatric patients with IHT. These observations indicate that they are separate entities that possibly should be managed using separate guidelines to optimize the use of CT-scans.

The incidence of ED visits due to head trauma in our study was lower than those reported in studies from England, Wales, and the United States [6, 7]. These differences could be explained by differences in organization and funding of health care systems that lead to different thresholds for visiting EDs for minor injuries. Other reasons could be differences in safety awareness by parents and differences in mandatory safety measures between countries, leading to fewer and less severe traumas. Such safety measures include laws on blood alcohol concentration limits and laws requiring children to use seatbelts in the back seat of cars and wearing helmets while cycling [21].

The incidence of TBI is difficult to study because of its indistinct definition. Therefore, we decided to study the incidence of ICI since it has a clear and straightforward definition that makes comparisons between studies easier. The rate of ICI was lower in our study than those in previous international studies that reported the rate of TBI on CT, which is equivalent to our definition of ICI [4, 8]. The relatively low rate of ICI in the present study may to some extent be explained by differences in aims, definitions and inclusion criteria of the studies. The present study aimed to describe the epidemiology and the management of all children attending an ED due to head trauma. Other studies, such as the PECARN trials [4], aimed to derive CDRs to assist clinicians in the decision whether to perform head CT-scan, and therefore excluded children with trivial head traumas. Our choice to include all patients with head trauma, irrespective of the severity of the trauma, may have resulted in a relatively higher proportion of patients with minor head trauma.

We found that falls were the most common trauma mechanism in patients with IHT. This observation is consistent with results from previous international studies, except for some studies reporting MVA as a common trauma mechanism for TBI [5]. In the present study, MVA was the trauma mechanism in only 1.2% of patients with IHT; however, it was the most common trauma mechanism in patients with MHT. The discrepancies in trauma mechanisms between studies can be explained by the differences in inclusion criteria and variations in the overall incidence of MVA in different countries [22].

The frequencies of CT-scanning of the head and admissions to a hospital ward were lower than those reported in several previous studies [4, 8, 20]. The relatively high CT-scanning rate in these studies may to some extent be explained by the exclusion of patients with “trivial” head trauma from their study populations [4, 23]. An alternative explanation could be less liberal CT-scanning in the present study. Although the rate of CT-scans was comparably low in our study, it was still approximately eight times higher than the rate of ICI. The relatively high rate of negative CT-scans raises the question of whether it is possible to reduce the use of CT-scanning further as it is associated with a risk for future malignancies [9]. Hospital admission and clinical observation of the patients is an alternative and equally safe measure to CT-scans in cases deemed to have a low risk for ICI, but admission costs are higher than those of performing CT-scans and discharging patients early [24]. We argue that admission may be the ethically preferable option in cases where the risk for ICI is low but cannot be ruled out with certainty. One would expect the admission rate to be higher in our study to compensate for the relatively conservative use of CT-scans, but the admission rates were similar or lower compared to previous studies [4, 8, 20].

The rates of ICI and NI in the present study were lower than those reported in previous studies [4, 23]. A contributing factor to the differences could be that we included more patients with less severe trauma. It is also possible that the low rates of ICI observed in the present study may be due to the relatively low CT-scanning rates, where minor ICIs may have remained undiagnosed. Other possible reasons may be mandatory safety measures for children in Sweden leading to less severe injuries and the fact that the incidence of traffic accidents with and without injuries and fatalities is lower in Scandinavia compared with the rest of Europe and North America [22].

In comparison with children with IHT, the relative risks of undergoing CT-scans of the head to be admitted to a hospital ward or an ICU were significantly higher in patients with MHT. Similarly, the relative risk of ICI and NI was significantly higher in MHT patients. The higher frequency of CT-scans of the head is likely explained by the assumed higher levels of trauma energy that MHT patients commonly are exposed to. These observations suggest that children with MHT represent an entity distinct from that of patients with IHT. Consequently, to reduce the number of excessive CT-scans in patients with IHT, we believe it is important to have separate management strategies for children with MHT and IHT.

The retrospective nature of data collection is the most relevant limitation of the present study. There is a risk of information bias which prevents us from drawing more than cautious conclusions from this study. Despite our countermeasures, another limitation is using of five different data collectors and the associated risk for differences in interindividual data interpretation.

## Conclusions

The rates of CT-scans and ICIs in this study were lower than that of previous studies, but no ICIs requiring interventions or resulting in deaths were missed. MHT patients had significantly higher rates of CT-scans, hospital admissions and ICIs than patients with IHT, indicating that they are separate entities that need to be managed using different guidelines to optimize the use of CT-scans.

## Data Availability

Data will be made available upon request.

## Abbreviations

ATLS®: Advanced Trauma Life Support
CDR: Clinical Decision Rules
CI: Confidence intervals
CT-scan: Computed tomography scan
ED: Emergency departments
ICD: International classification of disease
ICI: Intracranial injury
ICU: Intensive care unit
IHT: Isolated head trauma
MHT: Head trauma in connection with multitrauma
MVA: Motor vehicle accidents
NI: Neurosurgical intervention
PECARN: Pediatric Emergency Care Applied Research Network
SNC: Scandinavian Neurotrauma Committee
TBI: Traumatic brain injury
